# Clinical Observation of Patients Undergoing Glioma Surgery under Propofol and Sevoflurane Anesthesia: A Retrospective Study

**DOI:** 10.1155/2022/4516537

**Published:** 2022-06-08

**Authors:** Junbiao Fang, Hongfa Wang, Weihua Zhang, Kaichuang Yang, Weiyu Wang

**Affiliations:** ^1^Department of Anesthesiology, Zhejiang Provincial People's Hospital, Affiliated People's Hospital, Hangzhou Medical College, Hangzhou, Zhejiang, China; ^2^Department of Neurosurgery, Zhejiang Provincial People's Hospital, Affiliated People's Hospital, Hangzhou Medical College, Hangzhou, Zhejiang, China

## Abstract

**Objective:**

To observe the effects of propofol and sevoflurane anesthesia on patients undergoing glioma surgery.

**Methods:**

192 patients with gliomas treated in our hospital from January 2016 to January 2021 were selected. All patients were randomly divided into observation group and control group. The observation group was given sevoflurane and the control group was given propofol. The clinical effects of the two groups were observed.

**Results:**

Comparison of clinical indexes related to intraoperative conditions between the two groups revealed that the time of anesthesia and extubation after operation in the observation group were shorter than those in the control group, and the difference was statistically significant (*P* < 0.05). The amount of intraoperative bleeding in the observation group was less than that in the control group, and the difference was statistically significant (*P* < 0.05). There was no significant difference in intracranial operation time, operation time, fluid volume, and urine volume between the two groups (P < 05). Comparing the recovery time of anesthesia between the two groups, the recovery time of orientation and the time of eye-opening in the observation group were significantly shorter than those in the control group (*P* < 0.05). Comparing the consciousness and cognitive function of the two groups, the OAAS score of the observation group after extubation, before leaving the operating room and 1 hour after extubation, was significantly higher than that of the control group (*P* < 0.05), and the MMSE score l h after extubation was significantly higher than that of the control group (*P* < 0.05). Comparing the incidence of postoperative complications between the two groups, the number of cases of restlessness, urinary infection, deep vein thrombosis, and hypertension in the observation group was lower than that in the control group, with statistical significance (*P* < 0.05).

**Conclusion:**

The anesthesia time and extubation time of the sevoflurane anesthesia group were shorter than that of the propofol anesthesia group, and the orientation recovery time and eye-opening time were shortened. The OAAS score of the sevoflurane anesthesia group was higher than that of the propofol anesthesia group after extubation, before extubation, and 1 hour after extubation. The probability of postoperative complications in the sevoflurane anesthesia group was lower than that in the propofol anesthesia group. Sevoflurane anesthesia may be more suitable for surgical induction of glioma patients than propofol anesthesia.

## 1. Introduction

Glioma, derived from the neuroepithelium, is the most common intracranial malignant tumor. The annual incidence rate is 4/100,000–5/100,000, accounting for 40%–50% of all intracranial tumors [1, 2]. Surgical resection is the most direct and effective method for the treatment of gliomas. Maximum resection of tumor lesions can effectively prolong the survival time of patients and improve the quality of life [3]. However, mechanical or ischemic factors such as surgical trauma, the influence of anesthetic drugs, ischemia-reperfusion injury, and other mechanical or ischemic factors will inevitably cause damage to the brain tissue around the tumor, which will have a negative impact on the recovery of 'patients' consciousness and cognitive function after operation [4].

The effects of different anesthetics on the tumor microenvironment of malignant tumor cells have been gradually found and have a close impact on the follow-up treatment of tumor patients and the formulation of rehabilitation programs [5, 6]. Sevoflurane, isoflurane, propofol, and dexmedetomidine are the most commonly used anesthetic drugs in clinical glioma surgery. Sevoflurane and isoflurane are commonly used inhalation anesthesia induction drugs, while propofol is the most common intravenous anesthetic [7]. Surgical resection of malignant tumors of the nervous system not only requires stable and reliable anesthesia for a long time but also needs to reduce the interference to the internal environment and the damage of cellular immunity [8], so as to reduce the possibility of malignant tumor spreading. Propofol has the advantages of quick effect, short action time, rapid recovery, and high quality, which are convenient for the timely evaluation of neurological function after neurosurgery. In addition, propofol has the effect of constricting cerebral vessels and brain protection [9, 10]. Sevoflurane has low blood gas partition coefficient, rapid induction, and awakening and does not significantly change intracranial pressure while increasing cerebral blood flow. It has been found that sevoflurane can inhibit the proliferation and metastasis of lung cancer cells in vitro [11]. The advantages and disadvantages of their application in neurosurgical intracranial tumor resection is a controversial topic in the field of neurosurgical anesthesia. McCredie et al. [4] conducted a meta-analysis of the effects of propofol and inhalational anesthetics on intracranial pressure and the incidence of postoperative complications in elective craniotomy. The results showed that there was no significant difference between them, but due to the quality of the literature and other reasons, the research evidence is not sufficient. This study conducted a retrospective study on patients undergoing elective glioma resection to explore the effects of propofol and sevoflurane anesthesia on glioma resection patients.

## 2. Materials and Methods

### 2.1. Study Design and Participants

192 patients with glioma were selected from January 2016 to January 2021 in our hospital. All patients were randomly divided into observation group (*n* = 96) and control group (*n* = 96). There were 42 males and 54 females in the observation group, aged 29–64 years, and 45 males and 51 females in the control group, aged 27–68 years. There was no difference in the condition, course of disease, and other general data of the selected patients (*P* > 0.05), which were comparable. Informed consent was obtained from all the subjects. The ethics committee of our hospital approved this research plan. All participants underwent a complete medical history examination and clinical examination.

### 2.2. Anesthetic Method

After entering the operating room, the patients in the two groups were routinely connected with ECG monitoring, venous access, right internal jugular vein catheterization, and radial artery catheterization. Anesthesia induction was performed after sufficient preoxygenation of 3 min. Etomidate 0.2 mg/kg, sufentanil 0.3 ug/kg, and rocuronium 0.6 mg/kg were used as induction regimens. The observation group used sevoflurane to maintain anesthesia, the initial concentration of sevoflurane was inhaled continuously, the range of adjustment was ±0.2%, the inhalation concentration was 0.6∼1.5 MAC, and the BIS value was maintained at 40–60 during operation. The control group used propofol to maintain anesthesia, and the initial dose of 3 mg/(kg·h) was continuously pumped with an adjustment range of ±0.5 mg/(kg·h). Both groups were continuously pumped with remifentanil 0.3∼0.4 ug/(kg·min), the adjustment range was ±0.05 ug/(kg·h), and HR and BP were kept within ±20% of the basic value. The fluid loss was calculated according to the drainage volume and urine volume and was supplemented with the same amount of Ringer's solution. There was no significant difference in the vital signs and intake between the two groups. The operation was performed by the same treatment group physician to minimize human error.

### 2.3. Observation Index

The general situation, preoperative consciousness, tumor diameter, WHO grade, and preoperative complications of the two groups were recorded. Intraoperative information was recorded, including anesthesia and operation time, intracranial operation time, intake and output, postoperative destination, and hospitalization time; during the follow-up period, the incidence of complications in each system was recorded, and the recovery time of anesthesia was recorded. The recovery time of orientation, the time of respiratory recovery, the time of eye-opening, the time of extubation, and the time of leaving the operating room were recorded in the two groups after anesthesia. The state of consciousness and cognitive function were evaluated as follows: the patients were evaluated after extubation, before leaving the operating room, and 1 hour after extubation. The OAAS score was used to evaluate the state of consciousness of the patients. The patient is fully awake, and quick response after calling his name is 5 points; the patient is slow to respond after calling his name, and slow speech speed is 4 points; the patient only responds after repeated calls with vague language and dull eyes is 3 points; the patient responds only when he is nudged is 2; 1 point for lethargy. The cognitive function of the patients was evaluated by the MMSE score, and the total score was 30 points through five aspects of time, place orientation, memory, calculation, and language ability.

### 2.4. Inclusion and Exclusion Criteria

The inclusion criteria were as follows: (1) primary glioma, which was confirmed by pathology after operation; (2) no mental illness and no long-term use of psychotropic drugs; (3) the age is not less than 18 years; (4) the patients understand the details of the study and participate actively, and all patients sign the informed consent form. The exclusion criteria were as follows: (1) women during lactation or pregnancy; (2) complicated with other serious medical complications; (3) those who received other treatment at the present stage; (4) unconsciousness or mental abnormality.

### 2.5. Statistical Analysis

The data were analyzed by SPSS25.0 statistical software, and the clinical data (measurement data) were expressed as mean ± standard deviation (*x* ± *S*). One-way ANOVA was used for comparison between groups. The paired *t*-test was used for intragroup comparison, the independent sample *t*-test was used for intergroup comparison, counting data were expressed as rate (%),the *χ*^2^ test was performed, and the difference was statistically significant (*P* < 0.05).

## 3. Result

192 patients with glioma were admitted to our hospital from January 2016 to January 2021. All patients were randomly divided into observation group (*n* = 96) and control group (*n* = 96). There were 42 males and 54 females in the observation group, with an average age of 38.3 ± 7.6 years, BMI: 24.85 ± 2.52, 45 males and 51 females in the control group, with an average age of 37.9 ± 8.8 years, BMI: 24.70 ± 2.73. There was no difference in the condition, course of disease, and other general data of the selected patients (*P* < 0.05), as shown in Table 1.

### 3.1. Comparison of Intraoperative Conditions Related Clinical Indicators between the Two Groups

The anesthesia time and postoperative extubation time in the observation group were shorter than those in the control group, with statistical significance. The intraoperative blood loss in the observation group was less than that in the control group, and the difference was statistically significant (*P* < 0.05). There were no significant differences in intracranial operation time, operation time, fluid intake, and urine volume between the two groups, as shown in Table 2.

### 3.2. Comparison of Anesthetic Recovery Time between the two Groups

The recovery time of orientation and the time of eye-opening in the observation group were significantly shorter than those in the control group, as shown in Table 3.

### 3.3. Comparison of Consciousness State and Cognitive Function between the Two Groups

The OAAS score of the observation group was significantly higher than that of the control group after extubation, before leaving the operating room, and 1 h after extubation, and the MMSE score of l h after extubation was significantly higher than that of the control group, as shown in Table 4.

### 3.4. Comparison of the Incidence of Postoperative Complications

The number of patients with agitation, urinary tract infection, deep vein thrombosis, and hypertension in the observation group were lower than those in the control group, the differences were statistically significant (*P* < 0.05). There was no significant difference in programs: intracranial hemorrhage, intracranial infection, cerebral infarction, cerebral hernia, new aphasia, epilepsy, and new new activity obstacle, kidney, gastrointestinal bleeding, liver function abnormalities, constipation, upper respiratory tract infection, pulmonary infection, new hair arrhythmia, and cardiac insufficiency between the observation group and the control group (*P* > 0.05), as shown in Table 5.

## 4. Discussion

Gliomas, caused by malignant transformation of the glial cells in the brain, are the most common primary intracranial tumors, accounting for about 46% of all intracranial tumors [12, 13]. At present, surgical treatment is still the first choice to improve neurological function and prolong the survival time of patients with gliomas, supplemented by postoperative radiotherapy, chemotherapy, or immunotherapy [14]. All kinds of external stimulation and stress are important factors affecting the prognosis of perioperative patients, and they are also closely related to the immune status of the body [15, 16]. The occurrence and development of a tumor means that great changes have taken place in the immune system of the body. Studies suggest that general anesthetics may have a significant effect on the immune system. Thus, it has an important influence on the treatment and prognosis of tumor patients. The resection of glioma has the advantages of severe trauma and a long time of anesthesia and operation, and most of the postoperative complications are closely related to the degree of destruction of nerve function caused by the tumor before operation and the process of anesthesia and operation [17, 18]. Therefore, it is of great significance to optimize the anesthetic scheme and reduce postoperative complications.

Propofol and sevoflurane are commonly used as general anesthetics in clinics. Intravenous and inhaled drugs constitute a major part of the anesthetic regimen used in neurosurgery [19]. Sevoflurane is a relatively new inhaler, which is widely used in neurosurgery because of its advantages such as rapid awakening, reducing brain metabolism, and maintaining carbon dioxide responsiveness [20]. Propofol intravenous anesthesia is also a common and widely accepted method of anesthesia because it has the advantages of reducing cerebral blood volume, reducing intracranial pressure, maintaining self-regulation, and vascular reactivity [21–23].

In this study, the clinical indicators related to the intraoperative conditions between the two groups were compared. The anesthesia time and extubation time in the observation group were shorter than those in the control group, and the difference was statistically significant (*P* < 0.05). The intraoperative blood loss in the observation group was less than that in the control group, and the difference was statistically significant (*P* < 0.05). There was no significant difference in intracranial operation time, operation time, fluid intake, and urine output between the two groups (*P* < 0.05). Meta-analysis showed that total intravenous anesthesia based on propofol in patients with malignant tumors compared with general anesthesia with volatile anesthetics in patients in the total intravenous anesthesia group showed a better overall survival rate [22]. Comparing the recovery time of anesthesia between the two groups, the recovery time of orientation and the time of eye-opening in the observation group were significantly shorter than those in the control group (*P* < 0.05). Comparing consciousness and cognitive function between the two groups, the OAAS score of the observation group was significantly higher than that of the control group after extubation, before leaving the operating room, and 1 hour after extubation, and the MMSE score l h after extubation was significantly higher than that of the control group. Comparing the incidence of postoperative complications between the two groups, the number of patients with agitation, urinary tract infection, deep vein thrombosis, and hypertension in the observation group were lower than those in the control group, the difference was statistically significant (*P* < 0.05); there was no significant difference in intracranial hemorrhage, intracranial infection, cerebral hernia, cerebral infarction, new aphasia, new epilepsy, new limb dysfunction, renal dysfunction, gastrointestinal hemorrhage, liver dysfunction, constipation, upper respiratory tract infection, pulmonary infection, new arrhythmia, cardiac insufficiency, and hypotension between the two groups (*P* < 0.05). Some researchers have proposed that propofol-based total intravenous anesthesia and sevoflurane-based combined intravenous-inhalation anesthesia can be safely used in craniotomy for glioma. The two anesthetic schemes had no significant effect on the intraoperative condition of the patients and the incidence of postoperative complications during hospitalization. Xing et al. [7] conducted a cohort study of 294 patients with high-grade gliomas according to the intraoperative use of propofol and sevoflurane. There was no significant difference in the progression-free survival rate and overall survival rate between the two groups, and the effect of the two anesthetic schemes on the long-term prognosis of gliomas was not significant. In terms of postoperative complications, the different effects of these two anesthetic schemes on the incidence of postoperative complications in patients with glioma resection have not been reported in China. Hussain's study and other studies have suggested that the most common side effect of propofol is injection pain, with an incidence of between 30 and 70 percent. The combination of opioids and propofol may prevent injection pain usually associated with propofol. Patients not having nausea and vomiting may be due to the antiemetic properties of propofol [24]. In future research, we will carry out multi-center research, carry out more analyses and research, and draw more reliable conclusions.

To sum up, the anesthesia time and extubation time of the sevoflurane anesthesia group are shorter than those of the propofol anesthesia group, and the recovery time of the directional force and the time of eye-opening are shortened. The OAAS score is higher than that of the propofol anesthesia group after extubation, before leaving the operating room, and 1 hour after extubation, and the probability of postoperative complications for patients is somewhat reduced. In summary, sevoflurane anesthesia may be more suitable for surgical induction of glioma patients than propofol.

## Figures and Tables

**Table 1:** Baseline characteristics of participants.

Characteristics	Observation group (*N* = 96)	Control group (*N* = 96)
Gender (male/female)	58/38	52/44
Age (*x* ± *S*, years)	38.3 ± 7.6	37.9 ± 8.8
BMI (*x* ± *S*, kg/m^2^)	24.85 ± 2.52	24.70 ± 2.73
ASA grade (II/III)	82/14	19/17
Preoperative state of consciousness (awake/drowsiness)	85/11	92/4
Tumor diameter (*x* ± *S*, cm)	4.62 ± 1.35	4.88 ± 1.54
WHO classification (high/low)	80/16	76/20
Preoperative complications [*n* (%)]	36 (37.5)	39 (40.6)

**Table 2:** Comparison of clinical indexes related to intraoperative condition between the two groups.

Project	Observation group (*N* = 96)	Control group (*N* = 96)	*t*	*P*
Anesthesia time (min)	332 ± 86	385 ± 127	3.386	0.001
Intracranial operating time (min)	146 ± 81	150 ± 55	0.400	0.689
Operation time (min)	316 ± 83	347 ± 115	1.012	0.157
Intraoperative bleeding volume (ml)	808 ± 457	993 ± 785	1.996	0.047
Liquid intake (ml)	3139 ± 856	2893 ± 1214	1.623	0.106
Urine volume (ml)	1351 ± 614	1336 ± 385	0.203	0.840
Postoperative extubation time (h)	1.26 ± 1.31	4.79 ± 2.87	10.963	<0.001

**Table 3:** Comparison of anesthesia recovery time between the two groups (*x* ± *S*, min).

Group	*n*	Directional force recovery time	Breathing recovery time	Eye-opening time	Extubation time	Time away from the operating room
Observation group	96	17.19 ± 5.25	12.37 ± 2.36	9.47 ± 3.31	14.73 ± 4.24	19.21 ± 4.22
Control group	96	23.59 ± 3.62	12.29 ± 3.25	14.55 ± 2.28	15.57 ± 2.17	20.25 ± 6.37
*t*		9.833	0.191	12.384	1.728	1.334
*P*		<0.001	0.849	<0.001	0.086	0.184

**Table 4:** Comparison of OAAS and MMSE scores between the two groups at different time points (*x* ± *S*, points).

Group	*n*	OAAS			MMSE		
		After extubation	Before leaving the operating room	One hour after extubation	After extubation	Before leaving the operating room	One hour after extubation
Observation group	96	2.84 ± 0.13	4.06 ± 0.46	4.46 ± 0.74	24.80 ± 4.70	25.22 ± 4.89	28.38 ± 5.53
Control group	96	2.13 ± 0.19	3.14 ± 0.39	4.03 ± 0.61	24.72 ± 4.55	25.11 ± 4.41	26.23 ± 4.48
*t*		30.217	14.947	4.393	0.120	0.164	2.960
*P*		<0.001	<0.001	<0.001	0.905	0.870	0.003

**Table 5:** Comparison of the incidence of postoperative complications.

Project	Observation group (*n* = 96)	Control group (*n* = 96)	*χ* ^2^	*P*
Brain edema (mild/moderate/severe)	30/38/28	25/31/40	0.424	0.588
Intracranial hemorrhage (%)	5 (5.2)	4 (4.2)	1.528	0.336
Intracranial infection (%)	7 (7.3)	13 (13.5)	0.715	0.551
Cerebral hernia (%)	5 (5.2)	7 (7.3)	1.328	0.617
Cerebral infarction (%)	8 (8.3)	12 (12.5)	1.514	0.662
Restlessness in the awakening period (%)	25 (26.0)	47 (48.9)	7.237	0.015
New aphasia (%)	8 (8.3)	10 (10.4)	1.661	0.163
New onset epilepsy (%)	7 (7.3)	12 (12.5)	0.528	0.421
New limb movement disorder (%)	13 (13.5)	21 (21.9)	0.082	0.824
Urinary tract infection (%)	18 (18.8)	39 (40.6)	13.312	0.004
Abnormal renal function (%)	15 (15.6)	21 (21.9)	1.873	0.325
Gastrointestinal bleeding (%)	9 (9.4)	11 (11.5)	2.100	0.381
Abnormal liver function (%)	10 (10.4)	13 (13.5)	0.232	0.601
Constipation (%)	14 (14.6)	17 (17.7)	5.424	0.101
Deep venous thrombosis (%)	13 (13.5)	25 (26.0)	7.635	0.001
Upper respiratory tract infection (%)	17 (17.7)	21 (21.9)	4.102	0.697
Pulmonary infection (%)	12 (12.5)	16 (16.7)	1.541	0.821
New arrhythmia (%)	10 (10.4)	15 (15.6)	1.507	0.622
Cardiac insufficiency (%)	12 (12.5)	11 (11.5)	1.579	0.323
High blood pressure (%)	10 (10.4)	22 (22.9)	10.310	0.001
Hypotension (%)	7 (7.3)	9 (9.4)	1.932	0.594

## Data Availability

The data used to support the findings of this study are available from the corresponding author upon request.
